# Supplementary material for: COmic: convolutional kernel networks for interpretable end-to-end learning on (multi-)omics data
Source: Bioinformatics. 2023 Jun 30;39(Suppl 1):i76–85. doi: 10.1093/bioinformatics/btad204 (PMC10311322; doi:10.1093/bioinformatics/btad204)
Supplement: btad204_Supplementary_Data [file btad204_supplementary_data.pdf]

COmic: Convolutional Kernel Networks for Interpretable End-to-End Learning on (Multi-)Omics Data
Supplementary Materials

1 Additional Examples of the Local Interpretation Abilities of Attention-Based COmic Models

We present additional examples of the local interpretation capabilities of our attention-based COmic models. Figure 1 **A** shows a set of 20 randomly selected patients from the GSE11121 cohort that were correctly predicted to have a metastasis free survival of more than five years. Figure 1 **B** shows all patients from the GSE11121 cohort that were correctly predicted to have a metastasis free survival of less then five years. Figure 1 **C** shows all patients from the GSE11121 cohort that were wrongly predicted to have a metastasis free survival of more than five years when their real metastasis free survival was less than five years. The order of the pathway attention weights are the same as in the corresponding figure in the main manuscript.

One can see that the group of correctly classified patients with a metastasis free survival of more than five years show a similar pattern with a relative high attention weight on many different pathways. On the other hand, patients that were correctly classified to have a metastasis free survival of less than five years show attention weights that are focused on a single pathways or two pathways at most. Furthermore, the focused pathways are always the same, hedgehog signaling (pathway 29) and androgen response (pathway 16). The hedgehog signaling pathway is known to be important in tumor metastasis [3]. Furthermore, the androgen receptor plays an important role in the breast development cycle and is also known to affect breast cancer development and metastasis [5, 1]. The focus of attention weights on these two pathways in patients with shorter metastasis free survival reflects the known facts about the role that both pathways play in this process. However, there is an interesting pattern that can be observed for patients with a metastasis free survival of less than five years that were wrongly classified to have a metastasis free survival above five years. While some have the clear focus on either hedgehog signaling or androgen response and, therefore, strongly suggest that they are simple wrongly classified patients, most of them shows similar attention weight patterns to patients with an actual metastasis free survival above five years. This could hint at the fact that the group of wrongly classified patients with strong attention weight similarities to patients with longer metastasis free survival exhibit a different mechanism that causes metastasis than correctly classified patients with short metastasis free survival. And this different mechanism is not learned by the model. In any case, the interpretation indicates that there are different groups of wrongly classified patients and these groups should be investigated further. These findings support our claim of the benefits of using intrinsically interpretable models, due to the fact that these different groups in wrongly classified patients can be easily noticed directly from the model without further polluting the prediction task with necessary assumptions and additional computations for *post-hoc* interpretation models.

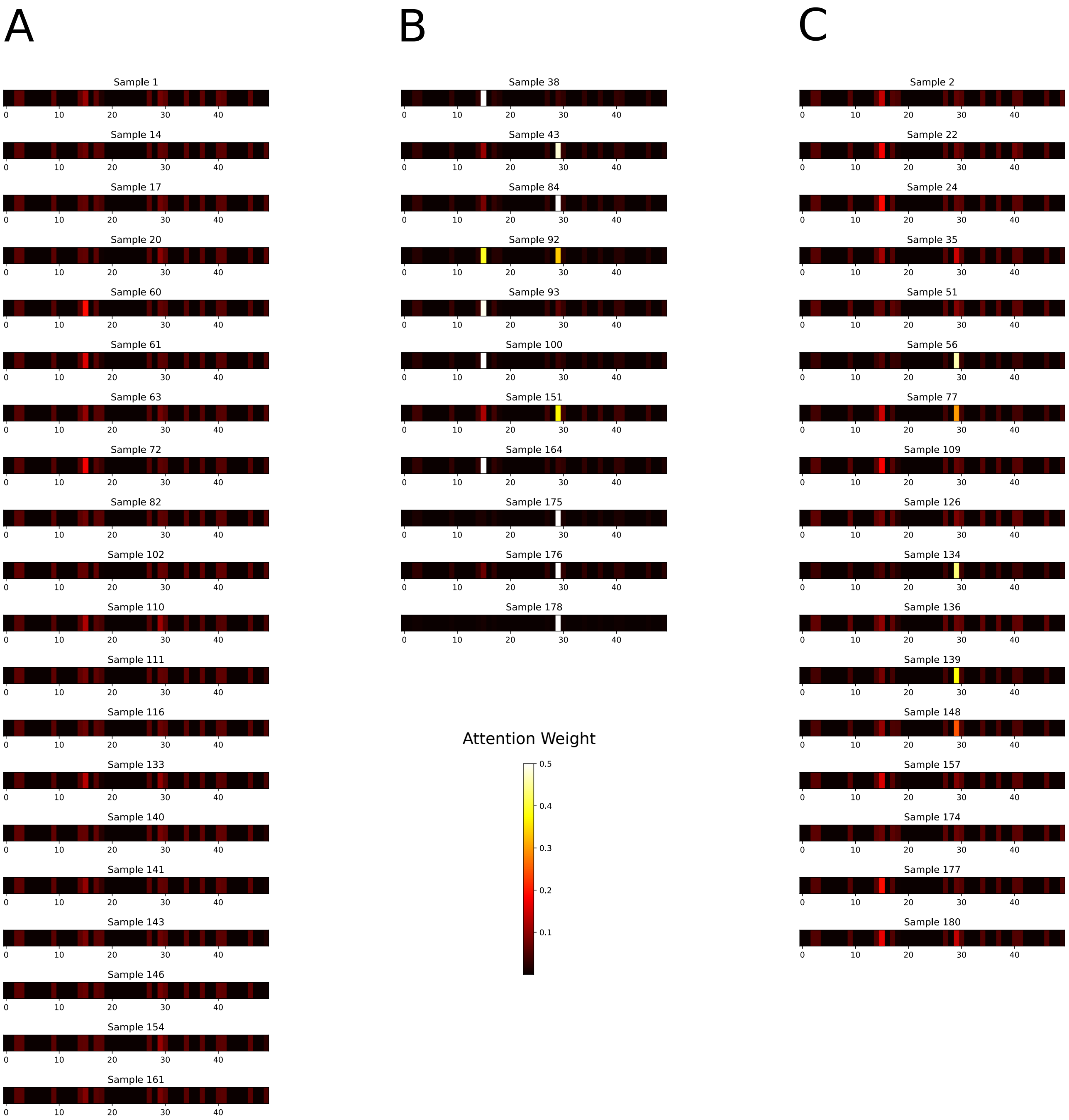

Figure 1: Additional examples of the local interpretation capability of attention-based COmic models. **A**: Randomly selected samples from the GSE11121 cohort that belong to patients that had a metastasis free survival of more than five years. All samples represent patients that were correctly predicted to have a metastasis free survival of more than five years. **B**: All samples from the GSE11121 cohort that belong to patients that had a metastasis free survival of less than five years and were correctly predicted to have a metastasis free survival of less than five years. **C**: All samples from the GSE11121 cohort that belong to patients that had a metastasis free survival of less than five years and were wrongly predicted to have a metastasis free survival of more than five years.

2 Societal and Environmental Impact

Medical data are notoriously biased against minorities and there are numerous examples of machine learning models that learn this biases and have a severe deterioration in performance with regard to minorities (see e.g., [4]). We did not include a statement on the societal impact of our work into the main manuscript due to the fact that we did not have meta information about ethnicity of patients included in the used benchmarks. Therefore, it was not feasible to investigate if the prediction performance and interpretation capabilities of COmic models change for minorities. However, we encourage researchers that want to apply COmic models on real-world data to investigate potential bias in their results.

All experiments were conducted using a single NVIDIA GeForce GTX 1080 Ti GPU. All experiments together required a approximated total of 146 hours of computing time. This resulted in total emissions of 15.77 kg CO_2e, which is equivalent to burning 7.9 kg of coal. To compensate this emissions, 0.26 tree seedlings have to sequester carbon for 10 years. These estimations were calculated using the Machine Learning Impact calculator[1] by Lacoste and colleagues [2].

References

[1] C. Arce-Salinas, M. C. Riesco-Martinez, W. Hanna, P. Bedard, and E. Warner. Complete response of metastatic androgen receptor-positive breast cancer to bicalutamide: Case report and review of the literature. *Journal of clinical oncology: official journal of the American Society of Clinical Oncology*, 34(4):e21–4, 2014.

[2] A. Lacoste, A. Luccioni, V. Schmidt, and T. Dandres. Quantifying the carbon emissions of machine learning. *arXiv preprint arXiv:1910.09700*, 2019.

[3] X. Li, W. Deng, C. D. Nail, S. K. Bailey, M. H. Kraus, J. M. Ruppert, and S. M. Lobo-Ruppert. Snail induction is an early response to gli1 that determines the efficiency of epithelial transformation. *Oncogene*, 25(4):609–621, 2006.

[4] J. Morley, C. C. Machado, C. Burr, J. Cowls, I. Joshi, M. Taddeo, and L. Floridi. The ethics of ai in health care: a mapping review. *Social Science & Medicine*, 260:113172, 2020.

[5] E. Pietri, V. Conteduca, D. Andreis, I. Massa, E. Melegari, S. Sarti, L. Cecconetto, A. Schirone, S. Bravaccini, P. Serra, et al. Androgen receptor signaling pathways as a target for breast cancer treatment. *Endocr Relat Cancer*, 23(10):R485–R498, 2016.

[1] https://mlco2.github.io/impact/
